# The Intention on Modern Contraceptive Use and Associated Factors among Postpartum Women in Public Health Institutions of Sodo Town, Southern Ethiopia 2019: An Institutional-Based Cross-Sectional Study

**DOI:** 10.1155/2020/9815465

**Published:** 2020-10-08

**Authors:** Natnael Atnafu Gebeyehu, Eyasu Alem Lake, Kelemu Abebe Gelaw, Gedion Asnake Azeze

**Affiliations:** ^1^Department of Midwifery, College of Health Science and Medicine, Wolaita Sodo University, Wolaita Sodo, Ethiopia; ^2^Department of Nursing, College of Health Science and Medicine, Wolaita Sodo University, Wolaita Sodo, Ethiopia

## Abstract

**Background:**

The postpartum period is an important transitional time for couples to put the decision on family planning utilization. However, women in Ethiopia are usually uncertain about the use of family planning during this period. This cross-sectional study was, therefore, aimed at determining the intention of modern contraceptive use and associated factors among postpartum women attending the immunization clinic in Sodo town, Ethiopia.

**Method:**

This institution-based cross-sectional study was conducted among 416 study participants from May 25 to June 20, 2019. The data were collected by using a systematic random sampling technique of interviewer-administered questionnaires. Descriptive analysis was done, and results were presented in texts and tables. Variables at bivariate analysis with a *p* value < 0.2 were moved to the multivariate logistic regression model to control potential confounding variables. Statistical tests at *p* value < 0.05 during multivariate analysis were considered as a cutoff point to determine statistical significance.

**Results:**

A total of 416 postpartum women participated in the study yielding a response rate of 98.1%. The prevalence of intention on modern contraceptive use among study participants was 70%. The odds of intention on modern contraceptive use was higher among study participants who had secondary school education (AOR = 2.052, 95% CI: 1.064-3.958), antenatal care visit (AOR = 1.74; 95% CI: 1.02-2.95), knowledge on modern contraceptive use (AOR = 2.54; 95% CI: 1.50-4.28), menses resumption (AOR = 2.05; 95% CI: 1.14-3.68), and husband approval to use contraceptives (AOR = 2.395, 95% CI: 1.501-5.458).

**Conclusion:**

The intention of modern contraceptive use among postpartum women was low. Family planning providers should emphasize reducing barriers of intention like lack of education, knowledge, male partner approval, antenatal care visit, and advise the impact of menses on fertility.

## 1. Introduction

Family planning is the intention, means, and methods that allows individuals to decide if and only when to have children. Access to safe, voluntary family planning is a human right [1]. This cost-effective public health and development intervention allows individuals to achieve desired birth spacing and family size and contributes to improved health outcomes for infants, children, women, and families [2].

Globally, in the year 2019, 1.1 billion reproductive age women need family planning, that is, they are either 842 million current users of modern contraceptives and 80 million used traditional methods whereas the remaining 190 million women want to avoid pregnancy but do not use any contraceptive methods. There is also a significant variation in terms of contraceptive prevalence rate among overall methods ranging from 4.6% in South Sudan to 72.1% in Canada [3].

Implementing postpartum family planning provision is an important strategy to reduce the unmet need for family planning. Postpartum women are among those with the greatest unmet need for family planning. Therefore, postpartum family planning focused on the prevention of unintended and closely spaced pregnancies through the first 12 months following childbirth [4]. The postpartum period provides an opportune movement when women should be counseled on birth spacing and family planning. Contraceptive options should be discussed and contraceptive methods should be provided if requested [5, 6].

The delivery of postpartum family planning services plays a pivotal role in reducing maternal mortality and morbidity. Multicountry studies have shown that accessing family planning can decrease maternal deaths by as much as 40%, infant mortality by 10%, and child mortality by 21% [7, 8]. The risk of maternal death increases as the number of children per woman rises four or more [9].

The London summit aimed to mobilize service deliveries to the rights of an additional 120 million women and girls in the world's poorest countries to use contraceptive without coercion or discrimination by 2020 [10]. Achieving this ambition target enables to prevent 100 million unintended pregnancies, 50 million abortions, 21,200 childbirth-related and maternal deaths, and 3 million infant deaths [11]. Ethiopia is one of the member states of the summit, working to achieve the targeted contraceptive prevalence rate of 55% by 2020 [12].

A study on postpartum contraceptive use among women in low- and middle-income countries in the first year after delivery was low, and desire for birth spacing and birth limiting was high [13]; yet, the use of postpartum family planning methods previously is low [14, 15], and risk of unintended pregnancy is high in the postpartum period [16–18].

In Ethiopia, the contraceptive prevalence rate among married women varied from 5% in the Somalia region to 53% in both the Amhara region and Addis Ababa. The overall modern contraceptive prevalence rate in the country is 40% [19]. The unmet need among women in the extended postpartum period is 86% [20].

A variety of works of literature among the general population showed that factors like shifting from one method of contraceptive to another [21], poor support from husband [22], maternal age [21], maternal education [21, 23, 24], positive attitude to contraceptive use [23], occupation [24], knowledge of contraceptives [22, 25], discussion on family planning with husband [21], myths and misconceptions regarding contraception [23], time of birth interval [22], joint fertility decision [24], and desire for live children [21] were some of the factors affecting the intention on contraceptive use among reproductive age women. However, the intention of contraceptive use in Ethiopia in general and in the study area in particular among postpartum women had not studied extensively.

Therefore, this study is aimed at assessing the intention of modern contraceptives use and associated factors among postpartum women in Sodo town, Ethiopia. The finding of this study could help the policymakers, programmers, planners, and different stakeholders on family planning issues in developing strategies to minimize closer birth intervals and to maximize postpartum contraceptive users for maternal and child wellbeing.

## 2. Methods and Materials

### 2.1. Study Area, Design, and Participation

An institution-based cross-sectional study was employed in Sodo town, Southern Ethiopia, from May 15 to June 10, 2019. Sodo town is located 320 km south of Addis Ababa, the capital city of Ethiopia. The town is divided into four administrative subcities. The total population of the town in 2018, received from the town administrative office, was 182,607 (93,139 males and 89,477 females). From these, 4963 were reproductive age group women (15-49 years). Among the reproductive age group, there were 893 postpartum women in the town. Functioning health facilities in the town includes two hospitals (one governmental and one private), three health centers, 17 medium and lower clinics, and 17 health posts.

All postpartum women who attended immunization clinics in Sodo town public health institutions were considered as a source of the population whereas all postpartum women attending immunization clinics in Sodo town public health institutions during the study period were the study population. Those postpartum women who attended the immunization clinic during the study period and selected with the sampling procedure were study units. All volunteer immunization clinic attendant postpartum women who were available during the study period were included in the study. However, postpartum women who were mentally and physically seriously ill during the study were excluded from the study.

### 2.2. Sample Size and Sampling Procedures

The sample size was determined by using a single population proportion formula by considering the following assumptions: 50% of the prevalence of intention on modern contraceptive use to gain a maximum sample size for the study, 95% confidence level, 5% marginal error, and 10% nonresponse rate. Based on these, a total of 424 immunization clinic attendant postpartum women were taken as a final sample size.

The total sample size was divided proportionally to the four health institutions based on their client flow. The proportionally allocated sample-sized was Wolaita Sodo teaching and referral hospital = 144, Sodo health center = 123, Gandaba health center = 95, and Geneme health center = 62. After reviewing the immunization registration book, the average flow of postpartum women attending the clinic for two months was taken to determine the sampling interval. Therefore, the average number of postpartum women attending immunization was 683 from all sites and yields the sampling interval of two. After selecting the first study participant by using the lottery method, we used a systematic sampling technique to recruit every *k*^th^ eligible respondents from the list of immunization registration serial numbers until the final sample size was reached through the proportional allocation of each clinic.

### 2.3. Operational Definitions

The intention of contraceptive use refers to the desire of all women who were found within twelve months postpartum period and who did not use any modern contraception during the time of the survey, but reporting that they want to use any of modern contraceptives at some future time.

Knowledge of modern contraceptive: knowledge index was built from the answers to 10 questions on modern contraceptives with Yes/No responses. After computing the mean score of knowledge questions, those who scored above the mean were considered as having good knowledge whereas those who scored below the mean score were considered as having poor knowledge.

Postpartum women are women who had live births within the past one year before the date of data collection.

Postpartum modern contraceptive use is when a postpartum woman reported actively using any modern contraception methods (pill, intrauterine device, injectable, condom (men or women), sterilization (men or women), or implants) during the 12 months following her most recent childbirth [15].

### 2.4. Data Processing and Analysis

The filled questionnaires were checked for completeness and entered into EpiData version 3.1 and exported to SPSS version 24 for statistical analysis. Descriptive statics were done and presented in the form of tables and texts. Bivariate and multivariate logistic regression models were used to identify associated factors. Those candidate variables at bivariate logistic regression with a *p* value < 0.2 were moved to the multivariate logistic regression model for the dependent variables to control potential confounding variables. Those variables with *p* value < 0.05 at multivariate analysis were considered as statically significant to the intention of modern contraceptive use in this study.

### 2.5. Data Collection Tool, Quality, and Procedures

A structured face-to-face interviewer-administered questionnaire was used to collect the data. The tool was prepared first in English and translated to the local language (Woliatagna) and then translated back to the English language to keep internal consistency. The tool was adopted from a similar study conducted in Aksum town [25]. The questioner contains sociodemographic characters, knowledge of modern contraceptive, maternal and reproductive health characteristics, and intention of future contraceptive use questions. Four diploma holder nurses and one bachelor of science (BSc.) degree holder health officers were recruited and trained for one day on how to interview and record the data before the start of the survey. A pretest was conducted on 42 postpartum women at Bodditi town health center. Data were checked for completeness and consistency by the supervisors and principal investigator.

### 2.6. Ethical Consideration

An ethical clearance letter was obtained from Wolaita Sodo University, College of Health Sciences, Department of Midwifery institutional review board. Written permission letters were obtained from the Sodo town health office. Participants were informed about the objectives of the study and reassured about the confidentiality of the findings. Written consent was obtained from each participant.

## 3. Results

### 3.1. Sociodemographic Characteristics of Study Participants

Among the total of 424 women expected to be included in the study, 416 had participated with the response rate of 98.1%. Of these, more than half, 249 (60%), were categorized under the age of 25-35 years. The mean ± standard deviation (SD) study participant's age was 25.84 ± 5.78. Wolaita were the predominant ethnic group, 290 (70%), followed by Gurage 97 (23%). Of the total study participants, the majority of them were protestant by religion 159 (38.2%) (Table 1).

### 3.2. Maternal and Reproductive Health Characteristics of the Study Participants

The majority of the study participants, 315 (75.7%), had two and above children during the time of the study. Nearly one-third of the respondents, 101 (24.3%), reported that they had only one child living with them. Two third (66.4%) of the study subjects had less than or equal to two living children. The majority of the respondents, 261 (62.7%), had antenatal care services. More than three-fourths of the study participants, 346 (83.2%), had received family planning counseling throughout their lifetime. Three hundred twenty-six (78.4%) of the study participants had a previous history of postnatal care following the visit. Slightly more than three-fourth, 316 (78%), the respondents had reported that they had a discussion postpartum family planning with their partners. The majority of postnatal women, 348 (83.7%), had husband approval of contraceptive, and 334 (80.3%) had a previous history of contraceptive use. Three hundred sixteen (76%) of the respondents said that menses had resumed after recent birth, and 333 (80%) explained that sexual activity had resumed. In the case of a decision on contraceptive use, less than half of the respondents (47.1%) reported that they had jointly put the decision to use contraceptives with their husbands (Table 2).

### 3.3. The Intention of Modern Contraceptive Use

More than two-third, 291 (70%), of the study participant expressed their intention to use modern contraceptives for the future. From those who desire to use contraceptive in the future, implants (125 (43%)) were the most intended contraceptive method followed by injectables (62 (21.3%)), pills (45 (15.5%)), condom (41 (14%)), IUCD (15 (5.2%)), and tubal ligation (3 (1%)). Concerning reproductive intention, the majority of the study participants, 164 (56.4%), had intended to use contraceptive for spacing, limiting (84 (28.9%)), want an additional child soon (37 (12.7%)), and undecided about future (6 (2%)). Half of the study participants, 146 (50.2%), planned timing of contraceptive use of intention was between six weeks to six months followed by within six weeks (67 (23%)), between six months to a year (42 (14.4%)), above a year (25 (8.6%)), and uncertain (11 (3.8%)). One hundred twenty-five (30%) of the study participants had no intention to use modern contraceptives in the future. The main reason for not using modern contraceptives in the future was husband refusal (56 (44.8%)), want an additional child (36 (28.8%)), fear of side effect (20 (16%)), and do not know about family planning (13 (10.4%)).

### 3.4. Factors Associated with Intention of Modern Contraceptive Use among Postpartum Women

On bivariate logistic analysis, participant's educational status, client's number of alive children, antenatal care follow-up, previous postnatal care follow-up, receiving counseling during antenatal care, discussion with a partner about postpartum family planning, menses status after childbirth, sexual activity after birth, previous history of contraceptive use, husband's approval of contraceptive use, and knowledge of postpartum contraceptive showed statistically significant association with intention of modern contraceptive use among postnatal women. On multivariate logistic analysis, participants who had a secondary level of education, attending ANC follow-up, menses status after birth, husbands' approval of family planning, and knowledge of postpartum family planning were found to have statistical significance to the intention of modern contraceptive utilization among postnatal women. Participants who had a secondary level of education were 2 times more likely to intend to use modern contraceptives than participants who had no formal education (AOR = 2.05; 95% CI: 1.06-3.96). Respondents who attended antenatal care service were 1.7 times more likely to intend to use modern contraceptives than their counterparts (AOR = 1.74; 95% CI: 1.02-2.95). The study subjects whose menses resumed were 2 times more likely intend to use than participants whose menses did not resume (AOR = 2.05; 95% CI: 1.14-3.68). Postpartum women who had husbands' approval of family planning use were 2.4 times more likely to intend to use modern contraceptives than postpartum women whose husband did not approve family planning use (AOR = 2.40; 95% CI: 1.50-5.46). Participants who had good knowledge about family planning were 2.5 times more likely to intend to use modern contraceptives than participants who had poor knowledge about family planning (AOR = 2.54; 95% CI: 1.50-4.29) (Table 3).

## 4. Discussion

The results showed that the prevalence of intention to use modern contraceptives among postpartum women was 70%. This finding is in line with that of a study conducted in Ghana where the magnitude of the intention of postpartum family planning was 70% [26]. This similarity might be due to the similarity of participants in the two studies in some sociodemographic characteristics. For instance, the average age of the participants in this study was 25.84 years, and in the Ghana study, it was 25.6 years. Moreover, the similarity of the study design between the two studies was also another reason.

In the present study, the most preferred modern contraceptives intended to use by study participants were implants (43%) and injectables 16%. The result was in line with the studies done in America [27] revealing that 38% and 12% of postpartum women preferred to use implants and injectables, respectively. There were also 19.2% of postpartum women who preferred to use implants whereas 18.4% of postpartum women intend to use injectables in Nigeria [28]. This might be due to the government's intention towards long-acting modern contraception provision among which affects the intention of modern contraceptive uses among participants in the future.

The findings of the current study were higher than studies done in Ethiopia on the intention of long-acting and permanent methods of contraception and associated factors among married women [21–24] and findings from studies done in Nigeria (64%, 65%) [28, 29]. The discrepancy was due to the difference between the type of study design used, and the time gap of the current study with these studies done in Ethiopia and Nigeria. In addition to this, the studies in Ethiopia were focused on long-acting and permanent methods that have low service users at a national level.

On the contrary, the finding of this study was also less than studies done in Ethiopia [25, 30], and America 90% [27]. The reason behind the lower magnitude of intention to use contraception was the difference in availability, accessibility, infrastructure, and socioeconomic status between the present study, and a study done in America. It was also because studies in Ethiopia were done on the major towns which enable clients to access adequate information and education as compared to the present study. This, in turn, contributes to the intention of using modern contraceptive use among study participants.

In the current study, multivariate analysis showed that women with secondary education were more likely to intend to use modern contraceptives in the future than women with no formal education. This study is inconsistent with the studies done in Adigrat town and Goba town [22, 31]. The finding was similar to studies done in Ethiopia [21, 23, 24, 33] and Uganda [32]. In EDHS 2019, 58% of women with more than secondary education are using any contraceptive method compared with 32% of women with no education [19]. The finding showed the importance of female education to enhance female decision-making on reproduction issues including family planning.

The findings of this study showed that postpartum women who had attended antenatal care were more likely to intend to use modern contraception than their counterparts. This finding was in agreement with studies done in Ethiopia [14], Kenya, and Zambia [33]. This might be due to the more the women attended the antenatal care visit during pregnancy, the more they heard and gained postpartum counseling which intern contributes to the intention of modern contraception after delivery.

In this study, postpartum women resumption of menses was more likely to intend to use modern contraception than those women whose menses was not resumed. The finding of the present study is consistent with studies conducted in Ethiopia [14, 15, 34], Malawi, and Kenya [35, 36]. The possible explanation was because most women perceived that the risk of pregnancy is related to only menses resumption and might not take family planning during the postpartum period.

Postpartum women who had good knowledge of contraceptives were more likely to intend to use modern contraceptives than women with poor knowledge of contraceptives. This finding is similar to studies done in Ethiopia [22, 25]. This could be due to the importance of knowledge on recognizing contraception which intern contributes to the intention of using contraceptive methods.

The current study revealed that postpartum women with husband approval of family planning were more likely to intend to use modern contraceptive than their counterparts. This finding is in agreement with studies done in Ethiopia [21, 25] and Ghana [26]. The possible explanation briefly indicated the importance of focusing on male involvement in family planning efforts because partners play a key role in deciding future contraceptive methods for their wives.

## 5. Conclusion

The finding of this study demonstrated that the intention of modern contraceptive use among postpartum women was low. It is therefore highly recommended that the intention of modern contraceptive use and initiation of family planning provision among postpartum women should become a standard of service delivery to achieve the 2020 goal set of Ethiopia as a country. Therefore, attending antenatal visits, maternal educational level, husband approval of family planning, resumption of menses after birth, and knowledge about modern contraceptives affect the intention of modern contraceptive use among postnatal women attending immunization clinics positively. Family planning providers should emphasize reducing barriers of intention like lack of education, knowledge, male partner approval, and antenatal care visit and advise the impact of menses on fertility.

## Figures and Tables

**Table 1:** Sociodemographic characteristics of postpartum women in the public health institution of Sodo town, Southern Ethiopia: 2019 (*n* = 416).

Variables	Intention to use contraception		Total (%)
	Yes Frequency (%)	No Frequency (%)	
Total	291 (70)	125 (30)	416 (100.0)
Age			
18-24	89 (30.6)	26 (20.8)	115 (25.0)
25-35	168 (57.7)	81 (64.8)	249 (60.0)
≥36	34 (11.7)	18 (14.4)	52 (15.0)
Ethnicity			
Wolaita	188 (64.6)	102 (81.6)	290 (70.0)
Gurage	81 (27.8)	16 (12.8)	97 (23.0)
Others	22 (7.6)	7 (5.6)	29 (7.0)
Religion			
Protestant	87 (29.9)	72 (57.6)	159 (38.2)
Orthodox	76 (26.1)	36 (28.8)	112 (26.9)
Muslim	98 (33.7)	11 (8.8)	109 (26.2)
Catholic	30 (10.3)	6 (4.8)	36 (8.7)
Marital status			
Married	200 (68.7)	95 (76.0)	295 (70.9)
Widowed	22 (7.6)	6 (4.8)	28 (6.7)
Living together	24 (8.2)	7 (5.6)	31 (7.5)
Divorced	45 (15.5)	17 (13.6)	62 (14.9)
Educational status			
No formal education	93 (32.0)	24 (19.2)	117 (28.1)
Primary education	75 (25.8)	37 (29.6)	112 (27.0)
Secondary education	76 (26.1)	46 (36.8)	122 (29.3)
Tertiary education	47 (16.2)	18 (14.4)	65 (15.6)
Partner's education status			
No formal education	94 (32.3)	29 (23.2)	123 (29.6)
Primary education	82 (28.2)	36 (28.8)	118 (28.4)
Secondary education	60 (20.6)	31 (24.8)	91 (22.0)
Tertiary education	55 (18.9)	29 (23.2)	84 (20.0)
Occupation			
Government worker	47 (16.2)	39 (31.2)	86 (20.7)
Private worker	98 (33.7)	48 (38.4)	146 (35.1)
Farmer	81 (27.8)	17 (5.8)	98 (23.6)
Housewife	65 (22.3)	21 (16.8)	86 (20.7)
Estimated monthly income			
<600 ETB	7 (2.4)	17 (13.6)	24 (6.0)
601-1550 ETB	187 (64.3)	71 (56.8)	258 (62.0)
1551-3200 ETB	64 (22.0)	26 (20.8)	90 (22.0)
>3201 ETB	33 (11.3)	11 (8.0)	44 (10.0)

**Table 2:** Maternal and reproductive health characteristics of postpartum women in the public health institution of Sodo town, Southern Ethiopia: 2019 (*n* = 416).

Variables	Intention to use contraception		Total (%)
	Yes Frequency (%)	No Frequency (%)	
Total	291 (70)	125 (30)	416 (100.0)
Number of alive children			
One	62 (21.3)	39 (31.2)	101 (24.3)
Two and above	229 (78.7)	86 (68.8)	315 (75.7)
Age of interval between children			
1 year	95 (32.6)	36 (28.8)	131 (31.5)
2 year	88 (30.2)	47 (37.6)	135 (32.5)
3 year	67 (23.0)	32 (25.6)	99 (23.8)
≥4 year	41 (14.2)	10 (8.0)	51 (12.3)
Attending antenatal care			
Yes	200 (68.7)	61 (48.8)	261 (62.7)
No	91 (31.3)	64 (51.2)	155 (37.3)
Receiving family planning counseling			
Yes	259 (89.0)	87 (69.6)	346 (83.2)
No	32 (11.0)	38 (30.4)	70 (16.8)
Postnatal care			
Yes	249 (85.6)	77 (61.6)	326 (78.4)
No	42 (14.4)	48 (38.4)	90 (21.6)
Discussion with partner on PPFP^a^			
Yes	230 (79.0)	86 (68.8)	316 (76.0)
No	61 (21.0)	39 (31.2)	100 (24.0)
Husband approval of contraception use			
Yes	262 (90.0)	86 (68.8)	348 (83.7)
No	29 (10.0)	39 (31.2)	68 (16.3)
Previous contraceptive use			
Yes	255 (87.6)	79 (63.2)	334 (80.3)
No	36 (12.4)	46 (36.8)	82 (19.7)
Menses status			
Resumed	243 (83.5)	73 (58.4)	316 (76.0)
Not resumed	48 (16.5)	52 (41.6)	100 (24.0)
Resumption of sexual activity Yes	242 (83.2)	91 (72.8)	333 (80.0)
No	49 (16.8)	34 (27.2)	83 (20.0)
Who decides to use family planning			
Wife	103 (35.4)	38 (30.4)	141 (33.9)
Husband	114 (39.2)	82 (65.6)	196 (47.1)
Together	74 (25.4)	5 (4.0)	79 (19.0)

^a^Postpartum family planning.

**Table 3:** Multivariate analysis of factors associated with intention to use modern contraceptives in the future among postpartum women in the public health institutions of Sodo town, Southern Ethiopia: 2019 (*n* = 416).

Variables	Intention to use contraception		AOR^a^ (95% CI)	COR^b^ (95% CI)	*p* value
	Yes Frequency (%)	No Frequency (%)			
Educational status					
No formal education	93 (79.5)	24 (20.5)	1	1	
Primary education	75 (67.0)	37 (33.0)	1.92 (1.05, 3.47)	1.65 (0.84, 3.21)	0.17
Secondary education	76 (62.3)	46 (37.7)	2.35 (1.31, 4.19)	2.05 (1.06, 3.96)	<0.001
Tertiary education	47 (72.3)	18 (27.7)	1.48 (0.73, 3.00)	1.47 (0.66, 3.29)	0.07
Number of alive children					
One	62 (21.3)	39 (31.2)	1	1	
Two and above	229 (78.7)	86 (68.8)	1.68 (1.05, 2.68)	1.41 (0.62, 3.21)	0.34
Attending antenatal care follow-up					
Yes	200 (68.7)	61 (48.8)	1.41 (0.62, 3.21)	1.74 (1.02, 2.95)	0.01
No	91 (31.3)	64 (51.2)	1	1	
Attending postnatal care follow-up					
Yes	249 (85.6)	77 (61.6)	1.74 (1.02, 2.95)	1.70 (0.69, 4.16)	0.09
No	42 (14.4)	48 (38.4)	1	1	
Receiving counseling during antenatal care					
Yes	259 (89.0)	87 (69.6)	3.54 (2.08, 6.00)	1.10 (0.48, 2.51)	0.23
No	32 (11.0)	38 (30.4)	1	1	
Discussion with partner on PPFP^c^					
Yes	230 (79.0)	86 (68.8)	1.71 (1.07, 2.74)	0.70 (0.31, 1.58)	0.06
No	61 (21.0)	39 (31.2)	1	1	
Menses status					
Resumed	243 (83.5)	73 (58.4)	3.61 (2.25, 5.78)	2.05 (1.14, 3.68)	<0.001
Not resumed	48 (16.5)	52 (41.6)	1	1	
Resumes sexual activity					
Yes	242 (83.2)	91 (72.8)	1.85 (1.12, 3.04)	0.77 (0.39, 1.53)	0.07
No	49 (16.8)	34 (27.2)	1	1	
Previously used contraceptive					
Yes	255 (87.6)	79 (63.2)	4.12 (2.49, 6.83)	1.62 (0.71, 3.71)	0.45
No	36 (12.4)	46 (36.8)	1	1	
Husband approval of contraceptives					
Yes	262 (90.0)	86 (68.8)	4.10 (2.39, 7.02)	2.40 (1.50, 5.46)	0.03
No	29 (10.0)	39 (31.2)	1	1	
Knowledge on contraceptive					
Good knowledge	144 (49.5)	38 (30.4)	2.24 (1.44, 3.50)	2.54 (1.50, 4.28)	<0.001
Poor knowledge	147 (50.5)	87 (69.6)	1	1	

^a^Crude odds ratio; ^b^adjusted odds ratio; ^c^postpartum family planning.

## Data Availability

All data generated or analyzed during our study are available from the corresponding author on reasonable request.

## Abbreviations

ANC: Antenatal care
AOR: Adjusted odd ratio
EDHS: Ethiopian Demographic Health Survey
IUCD: Intrauterine contraceptive device
SPSS: Statically Package for Social Science
WHO: World Health Organization.
